# Supplementary material for: An opioid-like system regulating feeding behavior in C. elegans
Source: eLife. 2015 Apr 21;4:e06683. doi: 10.7554/eLife.06683 (PMC4427864; doi:10.7554/eLife.06683)
Supplement: Supplementary file 1. — Genes that affect the growth when knocked down by RNAi. DOI: http://dx.doi.org/10.7554/eLife.06683.021 [file elife06683s001.docx]

## Supplementary File 1. Genes that affect the growth when knocked down by RNAi

|  | **Gene** | **RNAi effect** |
| --- | --- | --- |
| NLP family | *nlp-2* | slow growth |
|  | *nlp-3* | grow faster than control |
|  | *nlp-12* | slow growth |
|  | *nlp-13* | grow faster than control |
|  | *nlp-14* | slow growth |
|  | *nlp-24* | slow growth |
|  | *nlp-34* | slow growth |
| INS family | *ins-11* | grow faster than control |
|  | *ins-34* | slow growth |
|  | *ins-39* | slow growth |

## 
