# Supplementary material for: An opioid-like system regulating feeding behavior in C. elegans
Source: eLife. 2015 Apr 21;4:e06683. doi: 10.7554/eLife.06683 (PMC4427864; doi:10.7554/eLife.06683)
Supplement: Supplementary file 2. — Primers for transgenic worms. DOI: http://dx.doi.org/10.7554/eLife.06683.022 [file elife06683s002.docx]

## Supplementary File 2. Primers for transgenic worms

| Allele | Description | Sequence |
| --- | --- | --- |
| *adEx2557* | nlp-24 F | atatatacaggcggatctcggcg |
|  | nlp-24 B | CAAGCTTGGCGTAATCATGGTCaccgagaagagctccaacga |
|  | mcherry F | GACCATGATTACGCCAAGCTTG |
|  | mCherry B | AAGGGCCCGTACGGCCGACTAGTAGG |
|  | mCherry B* | GGAAACAGTTATGTTTGGTATATTGGG |
| *adEx2561* | npr-17 5' F | acagcagatttgtacgtgatattcctg |
|  | 5' F* | atgttcaaagttccactcgaagagtg |
|  | 5' B | GTCGACCTGCAGGCATGCAAGCTTaagaaaagttggattatgattactc |
|  | GFP F | tgtttcgaatgatactaacataac |
|  | GFP B | TTTGTATAGTTCATCCATGCC |
|  | 3' F | CATGGCATGGATGAACTATACAAAaattttaaataataatttaaattaacggg |
|  | 3' B* | ataacatcaagcaggctatcgag |
|  | 3' B | gatagcctgcttgatgttatcagt |
| *adEx2586* | MCC-gpa-4 promoter F BamH | aaGGATCCgacagaagacagagactcgag |
| *adEx2588* | MCC-gpa-4 promoter B Not | aaGCGGCCGCgctgtgaaagcatcccat |
|  | MCC-NPR-17 cDNA F Xma | aacccgggATGTCTACAAATTTGGTGGACTATG |
|  | MCC-NPR-17 cDNA B Nhe | aagctagcTCAAAGAAAAGTTGGATTATGATTACTC |
| *adEx2587* | MCC-ges-1 promoter F BamH | aaGGATCCagaccatacggaaatagctgttag |
| *adEx2589* | MCC-ges-1 promoter B Not | aaGCGGCCGCgaaactgtaagacgcagacgaat |
|  | MCC-NPR-17 cDNA F Xma | aacccgggATGTCTACAAATTTGGTGGACTATG |
|  | MCC-NPR-17 cDNA B Nhe | aagctagcTCAAAGAAAAGTTGGATTATGATTACTC |
| *adEx2590* | nlp-24 F | atatatacaggcggatctcggcg |
| *adEx2591* | nlp-24 B | GTCGACCTGCAGGCATGCAAGCTTgagaaggagaagcttgttcattctg |
| *adEx2593* | GFP F | AAGCTTGCATGCCTGCAGGTCGAC |
| *adEx2596* | GFP B | AAGGGCCCGTACGGCCGACTAGTAGG |
| *adEx2597* | GFP B* | GGAAACAGTTATGTTTGGTATATTGGG |
| *adEx2592* | MCC nlp-24 2kb F | tcagaagtatgtcaaccattcctg |
|  | MCC nlp-24 promoter B | GTCGACCTGCAGGCATGCAAGCTTtctgaaacattcttaattggtaatatac |
|  | GFP F | AAGCTTGCATGCCTGCAGGTCGAC |
|  | GFP B | AAGGGCCCGTACGGCCGACTAGTAGG |
|  | GFP B* | GGAAACAGTTATGTTTGGTATATTGGG |
