# Supplementary material for: An opioid-like system regulating feeding behavior in C. elegans
Source: eLife. 2015 Apr 21;4:e06683. doi: 10.7554/eLife.06683 (PMC4427864; doi:10.7554/eLife.06683)
Supplement: Supplementary file 3. — Primers for RNAi. DOI: http://dx.doi.org/10.7554/eLife.06683.023 [file elife06683s003.docx]

## Supplementary File 3. Primers for RNAi

| Description | Sequence |
| --- | --- |
| MCC-flp-6-F | TTACCGGTTATGAACTCTCGTGGGTTGA |
| MCC-flp-6-B | AAGGTACCCGTCCGAATCTCATGTATGC |
| MCC-flp-11-F | TTACCGGTACTGAATAATGACTCAATTCTCTGC |
| MCC-flp-11-B | AAGGTACCTTAATGATGAATTCGCCTCAGG |
| MCC-flp-16-F | TTACCGGTTGATCCTTCAGTTGTCAACAG |
| MCC-flp-16-B | AAGGTACCAGCACATTTATTGCTCGAACG |
| MCC-flp-18-F | TTACCGGTTTAGACATGCAACGGTGGT |
| MCC-flp-18-B | AAGGTACCCTTGATACATGTCAGACTGGC |
| MCC-flp-19-F | TTACCGGTATTCATTGCACCGACATGT |
| MCC-flp-19-B | AAGGTACCAGGTGTATTGAACCATGTCTG |
| MCC-flp-32-F | TTACCGGTATTCTTACGACATGCTCTCCT |
| MCC-flp-32-B | AAGGTACCTCAGTAGAGCAGGACTGATG |
| MCC-ins-2-F | TTACCGGTACCATGAACGCTATAATCTTCTG |
| MCC-ins-2-B | AAGGTACCCGCATTGATATCATATGATACATTACG |
| MCC-ins-4-F | TTACCGGTGCTCCAAGAGAATGTTTTCATTC |
| MCC-ins-4-B | AAGGTACCAGGAATTGGTTGCACATGG |
| MCC-ins-10-F | TTACCGGTAGCACTTCTAACAATGTCACTG |
| MCC-ins-10-B | AAGGTACCTTATAAAACGGAGCAGCAGG |
| MCC-ins-13-F | TTACCGGTTCCTTACATCAAAGTGCACTC |
| MCC-ins-13-B | AAGGTACCCAATTGAGACGTTGTACATGG |
| MCC-ins-33-F | TTACCGGTATGGCGAATACCTGCTTAATCC |
| MCC-ins-33-B | AAGGTACCACAACAATAGGTTCTACTCATCC |
| MCC-ins-36-F | TTACCGGTAATGAACATAGGCAAATGTTCCA |
| MCC-ins-36-B | AAGGTACCATTTGGGCAGCATAATTCACG |
| MCC-nlp-9-F | TTACCGGTAGAGAGATGGATCGATTCGCC |
| MCC-nlp-9-B | AAGGTACCTGTTATCCGGCAACGTCAAAC |
| MCC-nlp-23-F | TTACCGGTAGTCCCACCTGATATCACTTTC |
| MCC-nlp-23-B | AAGGTACCTTACTCCGAGAAATCATTGCGG |
| MCC-nlp-25-F | TTACCGGTATGCAATCACTAATTGCGCT |
| MCC-nlp-25-B | AAGGTACCACACTTCCTCAGCTACCAAG |
| MCC-nlp-27-F | TTACCGGTATAGGCCATTACCAATTTGACC |
| MCC-nlp-27-B | AAGGTACCTATCGAATTTACTTTCCCCATCC |
| MCC-nlp-29-F | TTACCGGTATGATTTCAACCTCTTCAATTCTTG |
| MCC-nlp-29-B | AAGGTACCATCCTCCATACATTCCGCG |
| MCC-nlp-34-F | TTACCGGTAGAATCATGATTTCCGCCAAG |
| MCC-nlp-34-B | AAGGTACCCGTCAGCAGCAGAAATCTATTT |
| MCC-nlp-38-F | TTACCGGTATGCAGCTGATACACTTTATTGTT |
| MCC-nlp-38-B | AAGGTACCAACTTCGAAGTATCGTGATGGC |
| MCC-nlp-41-F | TTACCGGTATGCTGGGACTCGTTTGC |
| MCC-nlp-41-B | AAGGTACCGTAGCTTTGCTCCTCCACT |
| MCC-nlp-42-F | TTACCGGTATGAGAGTGCAAGTAGTCACA |
| MCC-nlp-42-B | AAGGTACCATGCAAAGCCCAAATCCTG |
| MCC-nlp-44-F | TTACCGGTAGTCTGACTGAGTTTTCTCAGT |
| MCC-nlp-44-B | AAGGTACCTAACGCGTCGCACACTTT |
| MCC-nlp-46-F | TTACCGGTTCTGGTACTGTTGGTTCTCG |
| MCC-nlp-46-B | AAGGTACCTATTTTCCGGGTCGAAGACC |
| MCC-N NLP-40 F | TTGCGGCCGCTGCTATCTTTTGTTGCGACAG |
| MCC-N NLP-40 B | AACTGCAGATGGAGCTCTAGAATCCTCATTG |
